# The Preparation of Roots for Crowning and Gold Crowns

**Published:** 1895-05

**Authors:** 


ARTICLE VIII.

### THE PREPARATION OF ROOTS FOR CROWNING AND GOLD CROWNS.

BY DR. CAMPBELL.

*Gentlemen:* I must explain why this subject has been brought forward, and also why I am introducing it. The Secretary, after making every effort to secure a paper for our first meeting, could meet with no response, and appealed to the President, my father, for help in his difficulty.

It occurred to him that a discussion on root filling and gold crowns would prove of interest to all, and, although I have nothing new in connection with either of these, yet it seemed to me the subject was in itself so comparatively new that a discussion of the whole process, from the preparation of the root to the completion of the operation, could not fail to be interesting and profitable to us all.

I do not know to what extent the crowning, molar and bicuspid teeth with gold is practised by members of the Society, but I would encourage those who have not done much of this kind of work to adopt it more readily, as I must say I consider this one of the greatest strides yet made in conservative dentistry.

In suitable cases, if the process is carefully gone about, success is almost certain, and many cases of roots, which not so very long ago would have been victims to the forceps, can now be made serviceable for years.

I will give you an idea to what extent we have carried on this practice for the last year, as showing our confidence in this method of practice. During 1893 we put in fifty-five gold crowns, and during the ten months of the present year we have put in seventy-three gold crowns. Of these we know of no failures.

When suggesting this subject to the Secretary, my father had nothing new to offer, but he believed that a conversation on this subject would be almost certain to elicit some valuable suggestions.

I will state first how we proceed with regard to treating devitalized pulps.

Let us take a typical case: A patient comes to us with a molar or bicuspid tooth, which has been filled with a large amalgam filling, and, owing to decay or from other causes, the filling has come out and the walls of the crown of the tooth have crumbled away, so that it would be impossible to insert another amalgam filling. Very likely the pulp has been inflamed, and is causing considerable pain. Of course, in such a case, the first thing to do is to apply arsenic, which I invariably use in the form supplied by the S. S. White Co., and described by them as the "devitalizing fibre." It contains arsenious acid, creosote, tannic acid, and morphia, incorporated in fibre, and we find it the most suitable form of using the drug. After drying the cavity a small shred is laid on the dentine nearest the pulp, and a sufficient quantity of thoroughly mixed oxyphosphate of zinc (about the consistency of cream) laid over the whole surface of the tooth. After forty-eight hours this filling can be removed, and if the pulp is sufficiently destroyed it can be removed at once and the root filled.

Generally, however, I find it better to remove the dentine over the pulp and apply a small dressing of mixture of tannin and glycerine in the pulp chamber, and again seal with oxyphosphate of zinc or gutta-percha.

A week afterwards the pulp can be extracted with less pain, and much more easily, as it generally comes away *en masse.*

In the case, however, of a pulp, which has been devitalized from natural causes (such as exposure, owing to carious dentine, etc.), and become septic, one must proceed in a very different manner.

We have tried several methods, but the one with which we have had best results is the process described by Dr. Emil Schreier, of Vienna, at the World's Columbian Dental Congress, held in Chicago last year, in his paper on the Dalium Natrium process. This consists as, of course, you all know, in taking a small quantity of a prepared mixture of sodium-patassium properly on a barbed broach and applying it into the centre of the root canal or canals. The metals form soaps with the fatty products of the septic pulp. (*Cosmos*, page 864.)

Some fibres of cotton are then rolled round an ordinary fine broach and saturated with water; this is pushed up into the canals several times, until the whole of their contents are removed. An antiseptic dressing (we invariably use a mixture of "Oil of Cinnamon," 1 part; "Carbolic Acid," 2 parts; "Wintergreen," 3 parts); saturated in cotton wool and then placed in the canals and sealed over with gutta-percha. About a week later this dressing is removed, and if the patient has been free from pain the meanwhile, the roots are filled. With regard to the filling of the roots (of course after using rubber-dam, etc., to exclude saliva), we dry the canals first with the hot air from an ordinary rubber syringe, then apply absolute alcohol, and lastly, insert the canal plugger heated to red heat. After the canal has been properly dried, we take some of the chloro-percha solution on an ordinary plugger and force it into the canal, using a pumping motion.

When we consider the chloro-percha has completely filled the canals, we insert into each a gutta percha cone, and heating the plugger slightly, press it home. We then ask the patient to let us know when he feels a sensation at the end of the root, and when this takes place we stop.

We then take a small piece of gutta percha, soften it, and, placing it in the pulp chamber, with a large burnisher press it home. This ensures the chloro percha being forced through the apical foramen and effectually sealing the canals.

The root being successfully filled, the next step is to remove all decayed dentine and to shape the walls of the root itself, and whatever part of the crown there is left standing by grinding it down by carborundum wheels and discs, so that no overhanging edges remain, and the walls are as nearly as possible perpendicular. This is very important, as it allows the measuring wire to come off easily.

Assuming that the root is now ready for taking the measure, the next step is to take a piece of fine binding wire annealed and place it round the root just below the gum margin, leaving the free ends at the buccal part of the root and twist them with a pair of flat pliers, until the root is tightly embraced by the wire.

Instead of grasping the binding wire by the pliers, two other instruments are frequently used, but, I think, the pliers are the most handy, and always use them.

The wire is now removed, care being employed not to change the shape obtained. Should there be any pain experienced in applying the wire, a five per cent. solution of cocaine may be applied to the gum round the root, and left there for a few minutes with advantage.

It is most necessary, of course, to see that the wire is adjusted all round the root, as there is generally a little bleeding; this is frequently difficult, and it is sometimes necessary to use a little cotton wool or lint to mop away the blood. Where the gum is exuberant, it is most conveniently removed by ethylate of sodium.

The wire is now taken to the workroom and given to the mechanical assistant who is to make the crown. We tell him the tooth to be crowned, and show him how the wire was removed from the mouth. The wire is now laid on a piece of soft wood and receives a smart blow with a hammer. The shape is left clearly marked on the wood.

The wire is now cut with scissors opposite the twisted end and straightened out. This gives the exact length of the circumference of the root.

A piece of twenty-two carat gold plate rolled out to No. 27 American gauge is now cut exactly the length of the wire—the two ends brought together and soldered. The parts of the band which correspond to the mesial and distal surfaces of the root are scalloped out, as the alveolar process rises higher there than on the buccal or palative (lingual) surface.

The whole of the lower margin is then bevelled so that it may adapt itself to any irregularities of the root. The band is then contoured with contouring pliers and shaped so that the margin next the gum corresponds to the imprint of the wire made previously on the wood. A little X is scratched to show the buccal surface.

This part of the process takes about half an hour. It is now taken to the operating room, where it is driven on the root by means of the crown-driver and a mallet, if these are necessary; generally it is easily placed on the root by means of hand pressure. If the band does not fit the root very tightly, we contour it a little and try it again, and so on until it has a firm hold of the root. I then ask the patient to occlude his teeth to see that the band is free from the opposing tooth or teeth, and take a piece of quite soft stent and place it into the hollow of the band and instruct the patient to close his teeth. I then mop it over with the edge of a napkin dipped in cold water to harden it quickly and remove the stent, and generally the bend comes away embedded in it. If it does not, however, it can be afterwards removed and placed in position in the stent. A model and bite are now made, and when the band is in position in the model a piece of soft wax is placed in the centre, and when hard is carved into the shape of the grinding surface of a molar or bicuspid tooth, as the case may be, so that it articulates with the bite. It has, of course, to be the thickness (size) of the plate (twenty-seven gauge) that it will be when replaced by gold. It is now placed in a soft composition of modeling clay called moldine, and a metal of low fusibility poured into it. In this, again, when hardened, is poured the same kind of metal, but just on the point of setting, and we thus get a die and counterdie on which to strike up the cap. This is done by using a piece of brown paper first as a pattern and getting the approximate size of the cap, and replacing this by a piece of gold of the same carat and thickness already mentioned. After the cap is got, the hollows made by the cusps are filled up with solder, and a catch of ordinary eighteen carat plate gold soldered on the under surface of the cap. It is now attached in its proper position in relation to the band, and soldered with No. 00 or 1 solder. It is then filled up and stoned. We prefer not to put a fine polish on these all-gold crowns, as the glitter of the gold is much more apt to catch the eye when the crown is inserted in the mouth, than if left dulled.

The root being dried, and kept dry by means of the saliva ejector and cotton rolls, a sufficient quantity of oxyphosphate of zinc to fill the interior of the crown is mixed very thin (about the consistency of cream) and poured into the hollow of the crown, which is then placed in the root, care being taken to ensure its being driven quite home.

The saliva must be excluded for about a quarter of an hour, and then the superfluous oxyphosphate of zinc, which has oozed out between the edge of the band at the root under the gum, must be removed by means of a probe or other suitable instrument. If the bite does not come exact, a little can easily be cut from the opposing tooth.

This completes the operation, which takes altogether from the beginning four or five hours—three-quarters of an hour operator's time, and three to four hours of the mechanic's. In first bicuspids, when an all-gold crown might be an objection, as in the case of ladies, we frequently finish the crown in the manner above described, and cut a square opening on the buccal wall, into which is fitted an ordinary porcelain tooth, The pins of these are soldered to the crown and the porcelain cut flush with the surface of the crown. This we find an exceedingly useful method, not materially interfering with its strength, while certainly improving its appearance.—*Transactions of the Odonto-Chirurgical Society of Scotland.*

